# Sociodemographic characteristics and comorbidities of older adults associated with Primary Health Care quality

**DOI:** 10.1590/0034-7167-2024-0517

**Published:** 2026-08-07

**Authors:** Claudia Maria Ferrony Rivas, Artur Vernier Stochero, Paulo Jaeder Moraes Cervi, Francielle Liz Monteiro, Clandio Timm Marques, Clarissa Bohrer da Silva, Êmilly Barcelos Petter, Naiana Oliveira dos Santos

**Affiliations:** I Universidade Franciscana. Santa Maria, Rio Grande do Sul, Brazil; II Universidade do Estado de Santa Catarina. Florianópolis, Santa Catarina, Brazil; III Universidade Federal de Santa Maria. Santa Maria, Rio Grande do Sul, Brazil

**Keywords:** Aged, Primary Health Care, National Health Strategies, Quality of Health Care, Cross-Sectional Studies., Anciano, Atención Primaria de Salud, Estrategias de Salud Nacionales, Calidad de la Atención de Salud, Estudios Transversales.

## Abstract

**Objectives:**

to assess Primary Health Care quality, through older adults’ perception, and to analyze its associations with sociodemographic characteristics and comorbidities.

**Methods:**

a cross-sectional study was conducted using the Primary Care Assessment Tool with older adults. Data were subjected to statistical analysis using the Statistical Package for the Social Sciences software, employing parametric tests (t and ANOVA with Tukey) after confirming normality via Kolmogorov-Smirnov, with significance at p<0.05. Essential attribute scores were associated with participant sociodemographic data and comorbidities.

**Results:**

249 older adults registered in 25 Family Health Strategy units participated in the study, allowing for the assessment of Primary Health Care essential attributes. The results indicated globally satisfactory scores for most of the attributes analyzed, with the exception of first contact accessibility and comprehensiveness, which showed performances below expectations. Statistical analyses revealed significant associations between sociodemographic variables (sex, age group, and income) and the presence of comorbidities with certain attributes, suggesting that these characteristics may influence the perception and use of offered services.

**Conclusions:**

the research results show that, from older adults’ perspective, Primary Health Care quality in general is satisfactory.

## RESUMEN

**Objetivoss:**

evaluar la calidad de la Atención Primaria de Salud a través de la percepción de la población adulta mayor y analizar su asociación con las características sociodemográficas y las comorbilidades.

**Métodos:**

se realizó un estudio transversal con adultos mayores utilizando el *Primary Care Assessment Tool*. Los datos se analizaron estadísticamente con el programa *Statistical Package for the Social Sciences*, empleando pruebas paramétricas (t y ANOVA con Tukey) tras confirmar la normalidad mediante la prueba de Kolmogorov-Smirnov, con un nivel de significación p<0,05. Las puntuaciones de los atributos esenciales se asociaron con los datos sociodemográficos y las comorbilidades de los participantes.

**Resultados:**

participaron en el estudio 249 adultos mayores registrados en 25 unidades de la Estrategia de Salud Familiar, lo que permitió evaluar los atributos esenciales de la Atención Primaria de Salud. Los resultados indicaron puntuaciones globalmente satisfactorias en la mayoría de los atributos analizados, con excepción del acceso al primer contacto y la integralidad, que mostraron resultados por debajo de lo esperado. Los análisis estadísticos revelaron asociaciones significativas entre las variables sociodemográficas (sexo, grupo de edad e ingresos) y la presencia de comorbilidades con ciertos atributos, lo que sugiere que estas características pueden influir en la percepción y el uso de los servicios ofrecidos.

**Conclusiones:**

los resultados de la investigación muestran que, desde la perspectiva de las personas mayores, la calidad de la Atención Primaria de Salud, en general, es satisfactoria.

## INTRODUCTION

The increase in the global older adult population results in challenges for healthcare systems, as population aging brings specific demands, such as an increase in the prevalence and severity of diseases, especially non-communicable chronic diseases (NCDs). In Brazil, strategies to improve healthcare for older adults are constantly being developed with the aim of ensuring that the health needs of this population group are met in a comprehensive and effective manner^(1)^.

Strategies translated into public health policies should consider the specific needs of older adults and the cultural and social context, aiming for quality in the services offered^(2)^. From this perspective, Primary Health Care (PHC), defined as a set of measures aimed at health promotion, disease prevention, diagnosis, treatment, rehabilitation, harm reduction, and health maintenance, plays a key role in the care of older adults^(1-3)^.

PHC is one of the operational structures of the Health Care Network. Through the Family Health Strategy (FHS), the Ministry of Health seeks to reorganize PHC and achieve the objectives of universal access, equity, and comprehensiveness, which correspond to the Brazilian Health System principles. FHS aims to expand healthcare for the population, focusing on the family and the community, providing better health conditions for its users^(4)^.

Considering the positive impact of FHS on people’s health and our health indicators, it is crucial to assess services, identifying their strengths and weaknesses. This information guides health management effectively^(5,6)^.

PHC can be assessed through essential attributes, categorized as first contact accessibility, longitudinality, comprehensiveness, and care coordination, and derived attributes, categorized as family orientation, community orientation, and cultural competence^(4)^. One of the instruments used to assess quality is the Primary Care Assessment Tool (PCATool), which was developed to measure the presence and extent of PHC essential and derived attributes. In Brazil, the PCATool has been adapted and validated for use in different PHC contexts, and is an important tool for managers and healthcare professionals to assess and improve the quality of services offered^(7)^.

Considering that studies assessing PHC through older adults’ perception are scarce in the scientific literature^(8,9)^, this research seeks to fill an important knowledge need for PHC qualification, contributing to the care offered to older adults.

Recent studies show that, although there are advances in the structure and expansion of the supply of public healthcare services, obstacles related to access and quality perceived by users still persist^(10,11)^. These limitations contribute to the recurring need for secondary care services and emergency care. In this context, the present research proposes to fill a relevant gap in the field, offering support for the improvement of PHC and for strengthening care practices aimed at older adults.

## OBJECTIVES

To assess PHC quality in the municipality of Santa Maria, Rio Grande do Sul (RS), through older adults’ perception, and to analyze its associations with sociodemographic characteristics and comorbidities.

## METHODS

### Ethical aspects

This study was submitted to a Research Ethics Committee. The ethical and legal precepts involving research with human beings were considered, according to Resolution 466/12^(12)^. Informed Consent was obtained from all individuals involved in the study in writing.

### Study design, period and place

This analytical cross-sectional study was developed in accordance with STrengthening the Reporting of OBservational studies in Epidemiology guidelines^(13)^. The investigation was carried out in the municipality of Santa Maria, located in the central region of the state of RS, Brazil, from July 2023 to March 2024. The municipality has 49 Basic Health Units (BHUs), divided into 24 BHUs with Primary Care teams and 25 BHUs of FHS, subdivided in different locations of the city, representing coverage of 25.5% of the territory^(14)^. Data collection took place in the 25 FHSs. Data collection was carried out in private rooms.

### Population or sample; inclusion and exclusion criteria

The study included 249 older adults registered with the FHSs in the municipality of Santa Maria, RS. The estimated population of older adults (60 years of age or older) in the municipality is 39,147. The study population consisted of 17,081 older adults registered with the 25 FHSs in the municipality^(14)^, with a mean of 683 older adults per territory. To arrive at this number, a purposive sampling of approximately ten older adults per FHS was chosen, considering the operational feasibility and logistical limitations for travel to the territories. Sample size was determined using sample size calculations for finite populations, allowing for a 5% sampling error at a 95% confidence level. Participants were older adults who had received at least one healthcare service visit in the last 12 months. Older adults who reported private services as their regular source of healthcare were excluded. Recruitment was conducted in the health units’ waiting rooms by approaching older adults registered with FHS teams. The interviews were conducted in spaces previously provided by the FHSs themselves, in order to guarantee participant privacy. The sample was non-probabilistic, encompassing all individuals available to participate in the study.

### Study protocol

Data collection was carried out by previously trained researchers using the PCATool-Brazil, adult version, and a sociodemographic information questionnaire containing the following data: sex; age group; marital status (single, married, widowed); and years of education. Income source was categorized as retirement, social benefit, family support, and other. Income was classified as <1 minimum wage, 1-2, 2-3, >3 minimum wages, and not reported. In addition to sociodemographic variables, self-reported information on comorbidities was collected through self-reporting by participants during the interview.

The PCATool is composed of 87 items, divided into ten components, related to essential and derived attributes. In this research, PHC essential attributes were assessed, aiming for a more robust and detailed analysis of PHC central attributes. Therefore, the following components were explored: first contact accessibility; longitudinality; care coordination; and comprehensiveness. PHC assessment instrument items were answered using a Likert scale with the following response options: definitely yes (value 4); probably yes (value 3); probably not (value 2); definitely not (value 1). Blank and do not know/do not remember responses have a value of 9^(7)^. According to the PCATool manual, values from 1 to 4 for each response were used to calculate the mean score for each component and essential attribute of PHC. Thus, after obtaining the values, the data for each component were transformed into a scale of 0 to 10, and the simple arithmetic mean of the values was calculated. In the case of items C9, C10, C11, and D11 of the instrument, values were reversed according to the manual. Blank responses and responses of 9 (do not know/do not remember) were transformed into 2 (probably not) when they reached 50% or more of items in a component. Scores were classified as high/satisfactory when the score was ≥ 6.6, and low/unsatisfactory when the score was < 6.6, indicating the presence and extent of PHC attributes in the assessed services^(7)^. The cut-off point was determined by the responses to the instrument’s items, which indicate the minimum presence of characteristics of services recognized as oriented towards PHC.

### Analysis of results and statistics

Information was digitized into a Microsoft Excel database, and data statistical analysis was performed using the Statistical Package for the Social Sciences version 25. Sociodemographic characteristics (sex, age group, marital status, years of education, source of income, and income) were presented as distributions in absolute and relative values (percentages), and the age of older adults, in years, was presented as mean and standard deviation. For inferential analysis, the normality of the scores for the seven PHC attributes in relation to sociodemographic variables and comorbidities was initially verified using the Kolmogorov-Smirnov test, indicating that the data follow a normal distribution. Therefore, in the analysis of these variables, the parametric t-test for independent data and Analysis of Variance with Tukey post-hoc test were used. Differences were considered statistically significant when p-value was less than 0.05.

## RESULTS

Of the total of 249 older adults linked to the 25 FHUs in the municipality, 62.2% (n=155) were female; 52.2% (n=130) of older adults were married; 73.1% (n=182) had 0 to 8 years of schooling; 52.1% (n=125) had a family income between one and two minimum wages, with 71.1% (n=177) having retirement income. The mean age of older adults was 69.7 ± 6.9 years, ranging from 60 to 90 years, with the highest percentage in the 60 to 69 age group (57.4%, n=143). The information presented in Table 1 below shows the frequency distribution of the sociodemographic characteristics of the older adults participating in the study, conducted in the municipality of Santa Maria.

**Table 1:** Frequency distribution of sociodemographic characteristics of older adults in the study, Santa Maria, Rio Grande do Sul, Brazil, 2024 (N=249)

Variables	Results
**Sex (n (%))**	
Female	**155 (62,2%)**
Male	94 (37,8%)
**Age (mean ± standard deviation)**	**69,7 ± 6,9**
**Age group**	
60 to 69 years	**143 (57,4%)**
70 to 79 years	81 (32,5%)
≥ 80 years	25 (10,1%)
**Marital status (n (%))**	
Single	24 (9,7%)
Married	**130 (52,2%)**
Widowed	65 (26,1%)
Divorced/separated	30 (12,0%)
**Length of study (n (%))**	
0 to 8 years	**182 (73,1%)**
9 to 12 years	44 (17,7%)
≥ 13 years	23 (9,2%)
**Source of income (n (%))** Retired Social benefit Family support Others	**177 (71,1%)** 12 (4,8%) 20 (8,0%) 40 (16,1%)
**Income (n (%))**	
< 1 minimum wage per person	65 (27,1%)
1 - 2 minimum wages per person	**125 (52,1%)**
2 - 3 minimum wages per person	37 (15,4%)
> 3 minimum wages per person	13 (5,4%)
NOT reported	9^*^

* *Value not included in the analysis.*

**Figure 1:**
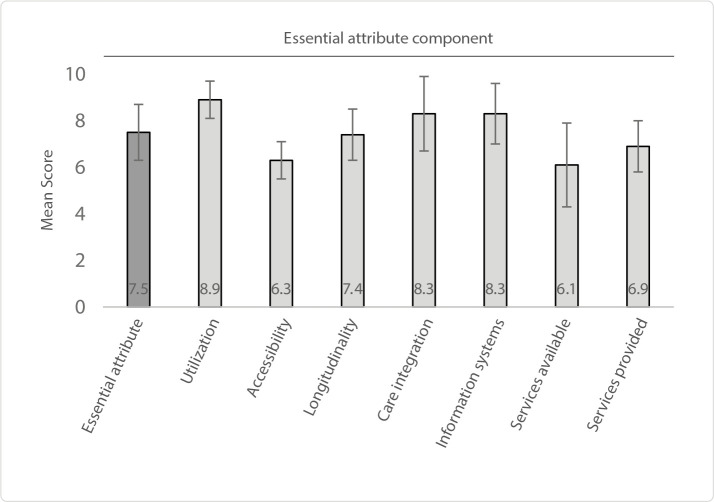
Comparison of mean scores for Primary Health Care essential attributes among older adults, Santa Maria, Rio Grande do Sul, Brazil, 2024 (N=249)

The overall mean score for assessing PHC essential attributes, from older adults’ perspective, was 7.5 ± 1.2, considered satisfactory (Figure 1). Assessing each essential attribute individually, satisfactory scores were obtained for first contact accessibility (utilization), longitudinality, coordination (care integration and information systems), and comprehensiveness (services provided). Conversely, unsatisfactory scores were obtained for first contact accessibility (accessibility) and comprehensiveness (services available) essential attributes. The data presented in Figure 1 illustrate a comparison of the mean scores of PHC essential attributes among older adults of the municipality of Santa Maria.

Based on the determination of the distribution of sociodemographic characteristics and the mean PHC essential attribute scores, an association was made among these variables (Table 2). A significant association was observed between the sex variable and accessibility (p=0.03), longitudinality (p=0.003) and services provided (p=0.028) essential attributes. These attributes were better assessed by older adult males, as observed in Table 2, due to their higher mean score. Concerning the age variable, the 60-69 age group (8.5 ± 1.3) showed a higher assessment of the information systems essential attribute compared to the other age groups (70-79 (8.4 ± 1.4) and 80 years or older (8.0 ± 1.4) (p=0.021)). Concerning family income, a significant association was observed with available services (p<0.001) and services provided (p=0.03) attributes. These attributes were rated higher by older adults earning more than three minimum wages and lower by older adults earning less than one minimum wage. The information presented in Table 2 below refers to the analysis of the association between sociodemographic variables and PHC attribute scores, considering the perspective of the older adults participating in the study in the municipality of Santa Maria.

**Table 2:** Analysis of the association between sociodemographic variables and Primary Health Care attribute scores from older adults’ perspective, Santa Maria, Rio Grande do Sul, Brazil, 2024 (N=249)

Variables	Attributes													
	Utilization	*p*	Accessibility	*p*	Longitudinality	*p*	Care integration	*p*	Information systems	*P*	Services available	*p*	Services provided	*p*
**Sex**														
Female	8.9 ± 1.5	0.455	6.2 ± 0.8	**0.030**	7.2 ± 1.2	**0.003**	8.3 ± 1.7	0.528	8.2 ± 1.4	0.456	6.1 ± 1.6	0.798	6.3 ± 1.6	**0.028**
Male	9.0 ± 1.6		**6.5 ± 1.**3		**7.7 ± 0.9**		8.5 ± 1.5		8.4 ± 1.3		6 ± 2.0		**6.8 ± 2.0**	
**Age**														
60 to 69 years	9.0 ± 1.5	0.322	6.2 ± 1.0	0.705	7.4 ± 1.1	0.873	8.5 ± 1.5	0.185	**8.5 ± 1.3**	**0.021**	6.2 ± 1.7	0.237	6.6 ± 1.8	0.412
70 to 79 years	9.0 ± 1.5		6.4 ± 1.1		7.4 ± 1.3		8.2 ± 1.7		8.0 ± 1.4		5.8 ± 1.9		6.2 ± 1.8	
≥ 80 years	8.5 ± 1.6		6.4 ± 0.5		7.3 ± 1.0		7.8 ± 1.4		8.0 ± 1.4		5.8 ± 1.7		6.5 ± 1.6	
**Education**														
0 to 8 years	9.0 ± 1.5	0.567	6.4 ± 1.1	0.692	7.3 ± 1.2	0.399	8.2 ± 1.5	0.206	8.2 ± 1.4	0.353	6.1 ± 1.8	0.610	6.5 ± 1.9	0.408
9 to 12 years	8.9 ± 1.5		6.2 ± 0.7		7.5 ± 0.8		7.5 ± 1.8		8.6 ± 1.1		6.1 ± 1.7		6.7 ± 1.5	
≥ 13 years	8.6 ± 1.8		6.3 ± 0.6		7.7 ± 0.8		9.1 ± 1.3		8.2 ± 0.9		5.7 ± 1.9		6.1 ± 1.5	
**Income (MW)**														
< 1 MW	8.9 ± 1.7	0.187	6.5 ± 1.2	0.215	7.2 ± 1.2	0.145	8.5 ± 1.4	0.755	8.2 ± 1.4	0.685	**5.3 ± 1.8^a^**	**< 0.001**	**5.9 ± 1.8^c^**	**0.030**
1-2 MWs	9.1 ± 1.4		6.3 ± 0.9		7.4 ± 1.2		8.3 ± 1.6		8.4 ± 1.4		6.3 ± 1.7		6.6 ± 1.8	
2-3 MWs	8.6 ± 1.8		6.1 ± 0.9		7.5 ± 0.9		8.7 ± 1.0		8.1 ± 1.3		6.1 ± 1.7		6.5 ± 1.6	
> 3 MWs	9.4 ± 1.2		6.4 ± 0.7		7.9 ± 0.9		7.8 ± 2.1		8.3 ± 1.3		**7.2 ± 1.9^b^**		**7.3 ± 1.5^c^**	

*MW - minimum wage; a - p = 0.034; b - p = 0.001; c - p = 0.048.*

In relation to the comorbidities self-reported by older adults in the PHC unit, it was observed that 54.7% (n=134) presented with hypertension (HT); 35.9% (n=88) had diabetes mellitus (DM); and 26.3% (n=64) had arthritis/osteoarthritis. Other comorbidities were reported, but with a lower percentage.

Based on the comorbidities presented by older adults assisted by PHC, an association was made with the mean scores of essential attributes. A significant association was observed between the presence of any comorbidity and the assessment of longitudinality (p=0.008) and information systems (p<0.001) attributes. Older adults with comorbidities rated these attributes better (7.5 ± 1) for longitudinality and 8.4 ± 1.3 for information systems. Table 3 shows the association between comorbidities and PHC attribute scores from older adults’ perspective in the municipality of Santa Maria.

**Table 3:** Analysis of the association between comorbidities and Primary Health Care attribute scores from older adults’ perspective, Santa Maria, Rio Grande do Sul, Brazil, 2024 (N=249)

Attributes	Variables							
	Comorbidity		Diabetes		Arthritis/osteoarthritis		HT	
	Yes	No	Yes	No	Yes	No	Yes	No
**Utilization**	9.0 ± 1.5	8.6 ± 1.7	9 ± 1.5	8.9 ± 1.6	9 ± 1.7	8.9 ± 1.5	**9.1 ± 1.4**	8.7 ± 1.7
***p***	0.131		0.814		0.812		**0.041**	
**Accessibility**	6.3 ± 0.9	6.7 ± 1.5	6.2 ± 0.7	6.4 ± 1.1	**6.1 ± 0.8**	6.4 ± 1.1	6.3 ± 0.9	6.4 ± 1.1
***p***	0.067		0.064		**0.047**		0.632	
**Longitudinality**	**7.5 ± 1.0**	6.8 ± 1.6	7.5 ± 0.9	7.3 ± 1.3	7.3 ± 1.3	7.4 ± 1.1	**7.7 ± 0.9**	7.1 ± 1.4
***p***	**0.008**		0.271		0.401		**< 0.001**	
**Care integration**	8.4 ± 1.6	8.1 ± 1.5	8.3 ± 1.8	8.3 ± 1.4	**7.8 ± 1.6**	8.5 ± 1.5	8.5 ± 1.6	8.1 ± 1.6
***p***	0.640		0.901		**0.021**		0.236	
**Information systems**	**8.4 ± 1.3**	7.5 ± 1.6	8.5 ± 1.2	8.2 ± 1.5	8.3 ± 1.6	8.3 ± 1.3	**8.7 ± 1.1**	7.8 ± 1.5
***p***	**< 0.001**		0.079		0.964		**< 0.001**	
**Services available**	6.0 ± 1.8	6.2 ± 1.7	5.6 ± 1.8	**6.4 ± 1.7**	6.4 ± 1.5	6 ± 1.8	6 ± 1.9	6.2 ± 1.6
***p***	0.684		**0.004**		0.082		0.351	
**Services provided**	6.5 ± 1.7	6.2 ± 2.0	6.4 ± 1.9	6.6 ± 1.7	6.5 ± 1.4	6.5 ± 1.9	6.4 ± 1.7	6.6 ± 1.8
***p***	0.362		0.351		0.870		0.654	

*HT - hypertension.*

Older adults with DM demonstrated lower scores on the available services (5.6 ± 1.8) attribute compared to people without DM (6.4 ± 1.7), with p = 0.004. Lower scores were also observed in older adults with arthritis on first contact accessibility (accessibility) (6.1 ± 0.8) and coordination (care integration) (7.8 ± 1.6) attributes, with p = 0.047 and p = 0.021, respectively. However, people with HT rated the services of first contact accessibility (utilization) (9.1 ± 1.4), longitudinality (7.7 ± 0.9), and coordination (information systems) (8.7 ± 1.1) more highly, with p = 0.041 for the first attribute and p < 0.001 for the others.

## DISCUSSION

The results of this study reveal, regarding the sociodemographic characteristics of research participants, a predominance of older adults who were female, aged between 60 and 90 years. There was a higher frequency of women aged 60 to 69, married, with low levels of education and low family income. These results are consistent with other national studies that address the characteristics of older adults using PHC^(15-17)^.

The mean scores for the attributes, in general, were considered satisfactory. However, the first contact accessibility essential attribute and its components, such as accessibility and usability, showed weaknesses, with a mean score slightly below the reference value in relation to accessibility. Accessibility in healthcare refers to structural aspects and ease of access to services, considering geographical and organizational factors, such as opening hours, appointment scheduling, and the use of communication tools^(4)^. It provides the population with efficient access to healthcare services on first contact^(18)^, guaranteeing timely care. The assessment of this attribute shows difficulties for users in accessing and using healthcare services. The utilization subdivision, which reported a high score, is linked to the use of the service by users for each new health problem and for each new episode of a previous problem^(7)^. Similar results were found in research conducted with adult users to assess PHC performance, with a high score for the utilization component^(19,20)^.

Through a high mean score, the presence of the longitudinality attribute in FHS teams was verified. A study developed to assess PHC quality in older adults’ perception supports these data, showing a satisfactory attribute assessment^(10)^. The longitudinality attribute implies the presence of a constant source of attention and its use over time, being crucial for strengthening an interpersonal relationship between users and the health team, reflecting mutual trust and bonding, promoting quality care that goes beyond the resolution of only immediate problems^(4)^.

A cross-sectional study involving adult PHC users in 32 municipalities of the 4^th^ Regional Health Coordination of Rio Grande do Sul (4^th^ RHC), using the PCATool, revealed an unsatisfactory mean score in the longitudinality attribute. However, it was observed that this attribute was better assessed in municipalities with smaller populations, lower Human Development Index, and greater coverage of FHS^(21)^. Santa Maria is the headquarters of the 4^th^ RHC, with an estimated population of 271,735 inhabitants^(22)^. In the municipality’s 2024 annual health program, there is an objective to expand coverage to 70% of the population through user registration by reference teams^(23)^. However, to date, no public information has been identified that confirms the effective implementation or achievement of this goal.

The care coordination essential attribute was considered high in both its components - care integration and information systems - coinciding with similar research conducted with 345 older adults, which obtained high scores in the analysis of results regarding care integration and information systems. Ribeiro and Cavalcanti^(24)^ postulate that coordinating care involves engaging in activities that guarantee the provision of individualized and comprehensive care, focusing on the continuity of the care process, and that obstacles faced by PHC in coordinating care include the need to overcome network fragmentation, the scarcity of specialist positions, the lack of integration of electronic health records, the lack of professional qualification, and the lack of recognition of the role of PHC by other healthcare services.

In the comprehensiveness attribute, divided into available services and services provided, the scores found were 6.9 and 6.1, respectively, a score considered satisfactory and slightly below the expected reference value. A study conducted in municipalities of RS, which sought to assess the comprehensiveness attribute in PHC services, showed unsatisfactory results in the attribute analysis. However, the available service component obtained a more positive assessment compared to the provided service component^(25)^. A systematic review of comprehensiveness in PHC in several countries revealed widespread dissatisfaction with the level of PHC orientation in this attribute, both in the services available and those provided. The importance of assessing the quality of services and adjusting and making available services more flexible to better meet demands and/or appropriately refer users is highlighted^(26)^. Furthermore, more significant support from healthcare institutions and their managers is recommended to improve physical, technological, and personnel infrastructure, including ongoing training for healthcare professionals^(26)^.

When calculating the essential score, which is the sum of the mean scores of the essential attributes divided by the number of components, we obtain a satisfactory value of 7.5, generally representing the presence and extent of essential attributes in the FHSs of the municipality. Research conducted in Manaus, Amazonas, using PCATool also revealed a high/satisfactory essential score^(27)^. However, reference values below the recommended levels were presented in studies that used the PCATool for PHC assessment, showing that not all healthcare services are guided by the essential attributes, requiring continuous assessments to improve healthcare in FHS services^(18,20,28)^.

When analyzing the association between sociodemographic variables and scores on essential attributes, it is observed that older adult males present better assessments regarding accessibility, longitudinality, and services provided, when compared to the mean responses of older women. These results differ from other similar studies, which indicate a better assessment of essential attributes by women^(29,30)^. A study on how male users rate first contact accessibility at PHC assessed this aspect as poorly oriented regarding accessibility, presenting a low mean score. However, the study does not address the assessment of attributes related to age group division^(31)^. Many older adults deal with chronic diseases, favoring more continuous contact with healthcare services^(32)^. This can strengthen the relationship with such services, increasing their familiarity and contributing to a positive attribute assessment and subdivisions mentioned.

The results show that improvements are needed, especially to enhance care for older women, who are the main users of health systems and have specific health needs^(33)^. Furthermore, the accessibility subdivision presented weaknesses overall, and especially regarding the accessibility of older women, it is necessary to guarantee quality access with services appropriate to their specific needs.

Older adults aged 60 to 69 years rated the coordination of information systems more positively compared to older adults aged 70 to 80 years or older. The information systems component proves to be accurate when users go to the healthcare service to present health records or information from a previous visit^(19)^.

It can be stated that longevity in Brazil is growing significantly, requiring special attention to this population. In this context, information systems play a crucial role in structuring and managing users’ health data, to facilitate the implementation of both individual and collective strategies^(34)^ and to make user information available throughout the service network. In other words, having a medical record containing their data at all points in the network is fundamental for healthcare coordination. Access to health information is a user’s right, and registration in the medical record is an essential part of this right^(35)^. Masochini *et al*.^(16)^ report that many older adults are still unaware of the possibility of accessing their medical records for consultation, demonstrating the importance of communicating this right to older adults.

Regarding family income, older adults with income equal to or greater than three minimum wages also demonstrated a better comprehensiveness attribute assessment. This result is similar to a previous study that used the same instrument, indicating a positive association between higher income and a better PHC assessment^(29)^. Another study found a positive association between access to initial contact and care coordination among older adults in vulnerable situations (low income, rural areas, and advanced age), suggesting significant progress in the pursuit of equity in access to and quality of healthcare services^(11)^. Therefore, efforts must be focused on making comprehensive care equitable for older adults, thus ensuring comprehensive care for the most economically vulnerable.

Most study participants had comorbidities, the most prevalent being HT, followed by DM and arthritis/osteoarthritis, which are prevalent NCDs among older adults in Brazil^(24)^. The analysis of the findings showed that older adults with comorbidities rated the longitudinality attributes and the information systems component more favorably. A positive assessment was also observed in a study with adult users, indicating that those with NCDs assess healthcare services more positively^(29)^. This research suggests that the favorable assessment stems from the greater use of these services by these users^(29)^. A study conducted in Belo Horizonte, Minas Gerais, presents divergent findings, showing the prevalence of NCDs associated with an unfavorable perception of PHC performance in the attributes^(30)^. It is highlighted that PHC plays a crucial role in the care of NCDs and in the control of risk factors, carrying out actions to prevent diseases, promote health and minimize harm.

This research found a significant association between older adults with HT and a better perception of aspects of PHC, such as the utilization component, the longitudinality attribute, and the information systems component. This finding corroborates a previous study conducted with adult users, which also highlighted a superior assessment of PHC by users with HT and DM^(29)^. However, other research indicates an association between older adults with HT and care coordination compared to older adults without this condition^(30)^. Furthermore, a study conducted in Fortaleza, Ceará, revealed that having HT or a mental health diagnosis may be associated with a lower perception of the comprehensiveness of available services, since people with chronic diseases, due to greater use of PHC, may be more exposed to negative experiences, which can result in a more critical perspective regarding the healthcare received^(20)^. Given that the aging population process has driven a significant increase in NCDs^(36,37)^, monitoring the occurrence of NCDs becomes indispensable for assessing effective public policies and improving health planning. This calls for a renewed approach in PHC, aiming to promote health, prevent avoidable deaths, and improve quality of life of affected patients^(36)^. Quality of care in PHC is essential to effectively address NCDs, improving the health and quality of life of the affected population.

In the study, most of results from the assessment of PHC essential attributes, as perceived by older adults, were satisfactory, with the exception of accessibility, which is a fundamental component for access to initial contact. Therefore, the importance of improving aspects such as adequate appointment times, efficient communication, offering alternative days, and organized appointment scheduling, among others, is highlighted. This reveals a challenge for the management of healthcare services and the need for adjustments to health teams’ work processes^(6)^.

### Study limitations

The instrument application was restricted to older adults who have access to FHS, excluding a significant portion of older adults who face barriers to access^(38)^. The participation of these individuals could offer distinct perspectives for assessing PHC essential attributes, especially regarding accessibility. Furthermore, the lack of specific studies on the use of the instrument with older adults^(39)^ hindered a more detailed analysis of the data found. It should also be noted that the use of a non-probabilistic sample compromises the representativeness of participants and may limit the generalization of results to other populations or similar contexts. It should also be considered that the instrument used was designed for application to adults in general without addressing the particularities relevant to older adults.

### Contributions to nursing, health, or public policy

The findings demonstrated by using the PCATool in assessing PHC among older adults may offer an opportunity to improve care for this population, enhancing the quality of healthcare for older adults in PHC. Furthermore, it contributes to improving scientific knowledge on the subject.

## CONCLUSIONS

This study assessed PHC quality from the perspective of 249 older adults registered in FHS units in the municipality of Santa Maria. The results indicated overall satisfactory scores for PHC essential attributes, with the exception of first contact accessibility and comprehensiveness, which showed lower performance. Statistically significant associations were identified between sociodemographic variables (sex, age, and income) and the presence of comorbidities with certain assessed attributes, suggesting that these characteristics influence perceived quality of care. Despite the overall positive assessment, challenges related to accessibility, comprehensiveness, and service provision persist, requiring strategic interventions. These findings highlight the need to consider the sociodemographic and clinical specificities of older adults in PHC planning and improvement, in order to promote more equitable and responsive care to their needs.

## Data Availability

The research data are available within the article.
