# Supplementary material for: Sociodemographic characteristics and comorbidities of older adults associated with Primary Health Care quality
Source: Rev Bras Enferm. 2026 Aug 7;79:e20240517. doi: 10.1590/0034-7167-2024-0517 (PMC13450830; doi:10.1590/0034-7167-2024-0517)
Supplement: Translation

# Translation: Características sociodemográficas e comorbidades da pessoa idosa associadas à qualidade da Atenção Primária à Saúde

**DOI:** 10.1590/0034-7167-2024-0517pt

**Authors:** Claudia Maria Ferrony Rivas, Artur Vernier Stochero, Paulo Jaeder Moraes Cervi, Francielle Liz Monteiro, Clandio Timm Marques, Clarissa Bohrer da Silva, Êmilly Barcelos Petter, Naiana Oliveira dos Santos

**Affiliations:** I Universidade Franciscana. Santa Maria, Rio Grande do Sul, Brasil; II Universidade do Estado de Santa Catarina. Florianópolis, Santa Catarina, Brasil; III Universidade Federal de Santa Maria. Santa Maria, Rio Grande do Sul, Brasil

**Keywords:** Pessoa Idosa, Atenção Primária à, Saúde, Estratégia Saúde da Família, Qualidade da Assistência à, Saúde, Estudos Transversais.

## Abstract

**Objetivos:**

avaliar a qualidade da Atenção Primária à Saúde, por meio da percepção da população idosa, e analisar suas associações com as características sociodemográficas e comorbidades.

**Métodos:**

estudo transversal, realizado por meio da aplicação do instrumento *Primary Care Assessment Tool* com pessoas idosas. Os dados foram submetidos à análise estatística no *software Statistical Package for the Social Sciences*, utilizando testes paramétricos (t e ANOVA com Tukey) após confirmação de normalidade via Kolmogorov-Smirnov, com significância p<0,05. As pontuações dos atributos essenciais foram associadas aos dados sociodemográficos e comorbidades dos participantes.

**Resultados:**

participaram do estudo 249 pessoas idosas cadastradas em 25 unidades da Estratégia Saúde da Família, permitindo a avaliação dos atributos essenciais da Atenção Primária à Saúde. Os resultados indicaram escores globalmente satisfatórios para a maioria dos atributos analisados, com exceção do acesso de primeiro contato e da integralidade, os quais apresentaram desempenhos aquém do esperado. Análises estatísticas revelaram associações significativas entre as variáveis sociodemográficas (sexo, faixa etária e renda) e a presença de comorbidades com determinados atributos, sugerindo que essas características podem influenciar a percepção e a utilização dos serviços ofertados.

**Conclusões:**

os resultados da pesquisa evidenciam que, na perspectiva da pessoa idosa, a qualidade da Atenção Primária à Saúde, de modo geral, apresenta-se como satisfatória.

## INTRODUÇÃO

O aumento da população de pessoas idosas mundial resulta em desafios para os sistemas de saúde, pois o envelhecimento populacional traz demandas específicas, como o aumento da prevalência e agravo de doenças, principalmente das doenças crônicas não transmissíveis (DCNTs). No Brasil, estratégias para a melhoria da atenção à saúde das pessoas idosas são constantemente elaboradas com o objetivo de garantir que as necessidades em saúde deste grupo populacional sejam supridas, de modo resolutivo e integral^(1)^.

As estratégias traduzidas em políticas públicas de saúde devem considerar as especificidades das pessoas idosas e o contexto cultural e social, visando à qualidade dos serviços ofertados^(2)^. Nesta perspectiva, a Atenção Primária à Saúde (APS), definida como um conjunto de medidas direcionadas à promoção da saúde, prevenção de doenças, diagnóstico, tratamento, reabilitação, redução de danos e manutenção da saúde, desempenha função primordial ao cuidado da pessoa idosa^(1-3)^.

A APS corresponde a uma das estruturas operacionais da Rede de Atenção à Saúde. Através da Estratégia Saúde da Família (ESF), o Ministério da Saúde busca reorganizar a APS e alcançar os objetivos da universalização, equidade e integralidade, que correspondem aos princípios do Sistema Único de Saúde. A ESF visa à expansão do cuidado à saúde da população, tendo como foco de atenção a família e a comunidade, proporcionando melhores condições de saúde aos seus usuários^(4)^.

Considerando o impacto positivo da ESF na saúde das pessoas e nossos indicadores de saúde, é crucial avaliar os serviços, identificando suas potencialidades e fragilidades. Essas informações orientam a gestão de saúde de forma eficaz^(5,6)^.

A APS pode ser avaliada através de atributos essenciais, categorizados em acesso ao primeiro contato, longitudinalidade, integralidade e coordenação da atenção, e de atributos derivados, categorizados em orientação familiar, orientação comunitária e competência cultural^(4)^. Um dos instrumentos utilizados para avaliar a qualidade é o *Primary Care Assessment Tool* (*PCATool*), que foi desenvolvido para medir a presença e extensão dos atributos essenciais e derivados da APS. No Brasil, o *PCATool* foi adaptado e validado para uso em diferentes contextos da APS, sendo uma ferramenta importante para gestores e profissionais de saúde avaliarem e melhorarem a qualidade dos serviços oferecidos^(7)^.

Considerando-se que estudos da avaliação da APS pela percepção das pessoas idosas são escassos na literatura científica^(8,9)^, esta pesquisa busca suprir uma necessidade de conhecimento importante para a qualificação da APS, contribuindo no cuidado ofertado às pessoas idosas.

Estudos recentes evidenciam que, embora haja avanços na estrutura e ampliação da oferta dos serviços públicos de saúde, ainda persistem entraves relacionados ao acesso e à qualidade percebida pelos usuários^(10,11)^. Essas limitações contribuem para a recorrente busca por serviços de atenção secundária e atendimentos de urgência. Nesse contexto, a presente pesquisa propõe suprir uma lacuna relevante no campo, oferecendo subsídios para a qualificação da APS e para o fortalecimento das práticas de cuidado destinadas à população idosa.

## OBJETIVOS

Avaliar a qualidade da APS, no município de Santa Maria, Rio Grande do Sul (RS), por meio da percepção da população idosa, e analisar suas associações com as características sociodemográficas e comorbidades.

## MÉTODOS

### Aspectos éticos

O presente estudo foi submetido à apreciação de Comitê de Ética em Pesquisa com Seres Humanos. Consideraram-se os preceitos éticos e legais que envolvem a pesquisa com seres humanos, conforme a Resolução n^**o**^ 466/12^(12)^. O Termo de Consentimento Livre e Esclarecido foi obtido de todos os indivíduos envolvidos no estudo por meio escrito.

### Desenho, período e local do estudo

Estudo transversal analítico, desenvolvido em conformidade com as diretrizes da ferramenta *STrengthening the Reporting of OBservational studies in Epidemiology* ^(13)^. A investigação foi realizada no município de Santa Maria, situado na região central do estado do RS, Brasil, durante o período de julho de 2023 a março de 2024. O município dispõe de 49 Unidades Básicas de Saúde (UBSs), divididas em 24 UBSs com equipes de Atenção Primária e 25 UBSs da ESF, subdivididas em diferentes locais da cidade, representando a cobertura de 25,5% do território^(14)^. A coleta de dados ocorreu nas 25 ESFs. Realizou-se a coleta em salas reservadas.

### População ou amostra; critérios de inclusão e exclusão

Participaram do estudo 249 pessoas idosas cadastradas nas ESFs do município de Santa Maria, RS. Estima-se que a população de pessoas idosas (60 anos ou mais de idade) do município é de 39.147 pessoas idosas. A população do estudo constitui-se de 17.081 pessoas idosas cadastradas nas 25 ESFs do município^(14)^, com média de 683 pessoas idosas por território. Para chegar a este número, optou-se por uma amostragem intencional de aproximadamente dez pessoas idosas por ESF, considerando-se a viabilidade operacional e as limitações logísticas para o deslocamento até os territórios. A amostra foi definida pelo cálculo amostral para populações finitas, admitindo-se um erro amostral de 5%, sob nível de confiança de 95%. Os participantes foram pessoas idosas que realizaram, ao menos, um atendimento no serviço de saúde nos últimos 12 meses. Foram excluídas pessoas idosas que referiram serviços particulares como fonte regular de atenção à saúde. O recrutamento foi realizado nas salas de espera das unidades, por meio da abordagem das pessoas idosas cadastradas nas ESFs. As entrevistas foram conduzidas em espaços previamente disponibilizados pelas próprias ESFs, de modo a garantir privacidade aos participantes. A amostra foi não probabilística, englobando as pessoas disponíveis para participar do estudo.

### Protocolo do estudo

As coletas foram realizadas por pesquisadores previamente capacitados, por meio da utilização do *PCATool*-Brasil, versão adultos, e um questionário de informações sociodemográficas contendo os seguintes dados: sexo; faixa etária; estado civil (solteiro, casado, viúvo); e tempo de estudo. A fonte de renda foi categorizada em aposentadoria, benefício social, apoio familiar e outros. A renda foi classificada em <1 salário mínimo, 1-2, 2-3, >3 salários mínimos e não informado. Além das variáveis sociodemográficas, foram coletadas informações autorreferidas sobre comorbidades, obtidas por meio de relato autorreferido pelos participantes durante a entrevista.

O *PCATool* é composto por 87 itens, divididos em dez componentes, relacionados aos atributos essenciais e derivados. Na presente pesquisa, avaliaram-se os atributos essenciais da APS, visando uma análise mais robusta e detalhada dos atributos centrais da APS. Sendo assim, foram explorados os seguintes componentes: acesso ao primeiro contato; longitudinalidade; coordenação de atenção; e integralidade. Os itens do instrumento de avaliação da APS foram respondidos utilizando escala Likert com as seguintes opções de resposta: com certeza sim (valor 4); provavelmente sim (valor 3); provavelmente não (valor 2); com certeza não (valor 1). Respostas em branco e não sei/não lembro possuem valor 9^(7)^. Conforme o manual do *PCATool*, valores de 1 a 4 de cada resposta foram utilizados para calcular o escore médio de cada componente e atributo essencial da APS. Assim, após a obtenção dos valores, os dados de cada componente foram transformados em escala de 0 a 10, realizando-se a média aritmética simples dos valores. No caso dos itens C9, C10, C11 e D11 do instrumento, os valores foram invertidos conforme o manual. Respostas em branco e respostas 9 (não sei/não lembro) foram transformadas em 2 (provavelmente não) quando atingiram 50% ou mais dos itens de um componente. Os escores foram classificados como alto/satisfatório quando escore foi ≥ 6,6, e baixo/insatisfatório, quando escore foi < 6,6, apontando a presença e a extensão dos atributos da APS nos serviços avaliados^(7)^. O ponto de corte foi determinado pelas respostas aos itens do instrumento, que indicam a presença mínima das características de serviços reconhecidos como orientados à APS.

### Análise dos resultados e estatística

As informações foram digitalizadas em um banco de dados do *Microsoft Excel*, e a análise estatística dos dados foi realizada pelo *software Statistical Package for the Social Sciences*, versão 25. As características sociodemográficas (sexo, faixa etária, estado civil, tempo de estudo, fonte de renda e renda) foram apresentadas por meio de distribuições em valores absolutos e relativos (percentuais), e a idade das idosas, em anos, foi apresentada em forma de média e desvio padrão. Para a análise inferencial, verificou-se, inicialmente, através do teste de Kolmogorov-Smirnov, a normalidade dos escores dos sete atributos da APS em relação às variáveis sociodemográficas e às comorbidades, indicando que os dados seguem uma distribuição normal. Portanto, na análise destas variáveis, foram utilizados os testes paramétricos t para dados independentes e Análise de Variância com post-hoc Tukey. As diferenças foram consideradas estatisticamente significativas quando o valor de p apresentou resultado inferior a 0,05.

## RESULTADOS

Do total de 249 pessoas idosas vinculadas às 25 ESFs do município, 62,2% (*n*=155) pertenciam ao sexo feminino; 52,2% (*n*=130) das pessoas idosas eram casadas; 73,1% (*n*=182) possuíam tempo de estudo de 0 a 8 anos; 52,1% (*n*=125) possuíam renda familiar entre um e dois salários mínimos, sendo 71,1% (*n*=177) com renda da aposentadoria. A idade média das pessoas idosas foi de 69,7 ± 6,9, variando entre 60 e 90 anos, com maior percentual na faixa etária de 60 a 69 anos (57,4%, *n*=143). As informações expostas na Tabela 1, apresentada a seguir, demonstram a distribuição das frequências das características sociodemográficas da população idosa participante do estudo, realizado no município de Santa Maria.

**Tabela 1:** Distribuição das frequências das características sociodemográficas da população de pessoas idosas do estudo, Santa Maria, Rio Grande do Sul, Brasil, 2024 (N=249)

Variáveis	Resultados
**Sexo (n (%))**	
Feminino	**155 (62,2%)**
Masculino	94 (37,8%)
**Idade (média ± desvio padrão)**	**69,7 ± 6,9**
**Faixa etária**	
60 a 69 anos	**143 (57,4%)**
70 a 79 anos	81 (32,5%)
≥ 80 anos	25 (10,1%)
**Estado civil (n (%))**	
Solteiro	24 (9,7%)
Casado	**130 (52,2%)**
Viúvo	65 (26,1%)
Divorciado/separado	30 (12,0%)
**Tempo de estudo (n (%))**	
0 a 8 anos	**182 (73,1%)**
9 a 12 anos	44 (17,7%)
≥ 13 anos	23 (9,2%)
**Fonte de renda (n (%))** Aposentado Beneficiário Familiar Outros	**177 (71,1%)** 12 (4,8%) 20 (8,0%) 40 (16,1%)
**Renda (n (%))**	
< 1 salário mínimo por pessoa	65 (27,1%)
1 - 2 salários mínimos por pessoa	**125 (52,1%)**
2 - 3 salários mínimos por pessoa	37 (15,4%)
> 3 salários mínimos por pessoa	13 (5,4%)
NÃO informado	9^*^

* *Valor não incluído na análise.*

A média geral dos escores de avaliação dos atributos essenciais da APS, sob a perspectiva da pessoa idosa, foi de 7,5 ± 1,2, considerada satisfatória (Figura 1). Avaliando, individualmente, cada um dos atributos essenciais, escores satisfatórios foram obtidos no acesso de primeiro contato (utilização), longitudinalidade, coordenação (integração de cuidados e sistemas de informação) e integralidade (serviços prestados). Em contrapartida, escores insatisfatórios foram obtidos nos atributos essenciais de acesso de primeiro contato (acessibilidade) e integralidade (serviços disponíveis). Os dados apresentados na Figura 1 ilustram uma comparação dos escores médios dos atributos essenciais da APS entre a população de pessoas idosas do município de Santa Maria.

**Figura 1:** Comparação das escores médios dos atributos essenciais da Atenção Primária à Saúde entre a população de pessoas idosas, Santa Maria, Rio Grande do Sul, Brasil, 2024 (N=249)

A partir da determinação da distribuição das características sociodemográficas e dos escores médios dos atributos essenciais da APS, realizou-se a associação entre essas variáveis (Tabela 2). Associação significativa foi observada entre a variável sexo e os atributos essenciais de acessibilidade (*p*=0,03), longitudinalidade (*p*=0,003) e serviços prestados (*p*=0,028). Esses atributos foram mais bem avaliados pelas pessoas idosas do sexo masculino, conforme observado na Tabela 2, pelo maior escore médio. Na variável idade, observou-se que a faixa etária de 60 a 69 anos (8,5 ± 1,3) apresentou maior avaliação no atributo essencial de sistemas de informação em relação às demais faixas (70-79 (8,4± 1,4) e 80 anos ou mais (8,0 ±1,4) (p=0,021)). Em relação à renda familiar, associação significativa foi observada com os atributos de serviços disponíveis (*p*<0,001) e serviços prestados (*p*=0,03). Esses atributos foram mais bem avaliados pelas pessoas idosas com mais de três salários mínimos e mais mal avaliados pelas pessoas idosas com menos de um salário mínimo. As informações apresentadas na Tabela 2, disposta a seguir, referem-se à análise da associação entre as variáveis sociodemográficas e os escores dos atributos da APS, considerando a perspectiva das pessoas idosas participantes do estudo no município de Santa Maria.

**Tabela 2:** Análise da associação entre as variáveis sociodemográficas e os escores dos atributos da Atenção Primária à Saúde sob a perspectiva da pessoa idosa, Santa Maria, Rio Grande do Sul, Brasil, 2024 (N=249)

Variáveis	Atributos													
	Utilização	*p*	Acessibilidade	*p*	Longitudinalidade	*p*	Integração de cuidados	*p*	Sistemas de informação	*P*	Serviços disponíveis	*p*	Serviços prestados	*p*
**Sexo**														
Feminino	8,9 ± 1,5	0,455	6,2 ± 0,8	**0,030**	7,2 ± 1,2	**0,003**	8,3 ± 1,7	0,528	8,2 ± 1,4	0,456	6,1 ± 1,6	0,798	6,3 ± 1,6	**0,028**
Masculino	9,0 ± 1,6		**6,5 ± 1,**3		**7,7 ± 0,9**		8,5 ± 1,5		8,4 ± 1,3		6 ± 2,0		**6,8 ± 2,0**	
**Idade**														
60 a 69 anos	9,0 ± 1,5	0,322	6,2 ± 1,0	0,705	7,4 ± 1,1	0,873	8,5 ± 1,5	0,185	**8,5 ± 1,3**	**0,021**	6,2 ± 1,7	0,237	6,6 ± 1,8	0,412
70 a 79 anos	9,0 ± 1,5		6,4 ± 1,1		7,4 ± 1,3		8,2 ± 1,7		8,0 ± 1,4		5,8 ± 1,9		6,2 ± 1,8	
≥ 80 anos	8,5 ± 1,6		6,4 ± 0,5		7,3 ± 1,0		7,8 ± 1,4		8,0 ± 1,4		5,8 ± 1,7		6,5 ± 1,6	
**Escolaridade**														
0 a 8 anos	9,0 ± 1,5	0,567	6,4 ± 1,1	0,692	7,3 ± 1,2	0,399	8,2 ± 1,5	0,206	8,2 ± 1,4	0,353	6,1 ± 1,8	0,610	6,5 ± 1,9	0,408
9 a 12 anos	8,9 ± 1,5		6,2 ± 0,7		7,5 ± 0,8		7,5 ± 1,8		8,6 ± 1,1		6,1 ± 1,7		6,7 ± 1,5	
≥ 13 anos	8,6 ± 1,8		6,3 ± 0,6		7,7 ± 0,8		9,1 ± 1,3		8,2 ± 0,9		5,7 ± 1,9		6,1 ± 1,5	
**Renda (SM)**														
< 1 SM	8,9 ± 1,7	0,187	6,5 ± 1,2	0,215	7,2 ± 1,2	0,145	8,5 ± 1,4	0,755	8,2 ± 1,4	0,685	**5,3 ± 1,8^a^**	**< 0,001**	**5,9 ± 1,8^c^**	**0,030**
1-2 SMs	9,1 ± 1,4		6,3 ± 0,9		7,4 ± 1,2		8,3 ± 1,6		8,4 ± 1,4		6,3 ± 1,7		6,6 ± 1,8	
2-3 SMs	8,6 ± 1,8		6,1 ± 0,9		7,5 ± 0,9		8,7 ± 1,0		8,1 ± 1,3		6,1 ± 1,7		6,5 ± 1,6	
> 3 SMs	9,4 ± 1,2		6,4 ± 0,7		7,9 ± 0,9		7,8 ± 2,1		8,3 ± 1,3		**7,2 ± 1,9^b^**		**7,3 ± 1,5^c^**	

*SM - salário mínimo; a - p = 0,034; b - p = 0,001; c - p = 0,048.*

Com relação às comorbidades autorreferidas pelas pessoas idosas da APS, observou-se que 54,7% (*n*=134) apresentavam hipertensão arterial sistêmica (HAS), 35,9% (*n*=88), diabetes *mellitus* (DM), e 26,3% (*n*=64), artrite/artrose. Outras comorbidades foram relatadas, porém com menor percentual.

A partir da determinação das comorbidades apresentadas pelas pessoas idosas atendidas pela APS, foi realizada associação com os escores médios dos atributos essenciais. Associação significativa foi observada entre a presença de alguma comorbidade e a avaliação dos atributos de longitudinalidade (*p*=0,008) e sistemas de informação (*p*<0,001). Pessoas idosas com comorbidades avaliaram melhor esses atributos (7,5 ± 1) para longitudinalidade e 8,4 ± 1,3 para sistemas de informação. A Tabela 3 mostra a associação entre comorbidades e os escores dos atributos da APS sob a perspectiva da pessoa idosa, no município de Santa Maria.

**Tabela 3:** Análise da associação entre as comorbidades e os escores dos atributos da Atenção Primária à Saúde sob a perspectiva da pessoa idosa, Santa Maria, Rio Grande do Sul, Brasil, 2024 (N=249)

Atributos	Variáveis							
	Comorbidade		Diabetes		Artrite/artrose		HAS	
	Sim	Não	Sim	Não	Sim	Não	Sim	Não
**Utilização**	9,0 ± 1,5	8,6 ± 1,7	9 ± 1,5	8,9 ± 1,6	9 ± 1,7	8,9 ± 1,5	**9,1 ± 1,4**	8,7 ± 1,7
***p***	0,131		0,814		0,812		**0,041**	
**Acessibilidade**	6,3 ± 0,9	6,7 ± 1,5	6,2 ± 0,7	6,4 ± 1,1	**6,1 ± 0,8**	6,4 ± 1,1	6,3 ± 0,9	6,4 ± 1,1
***p***	0,067		0,064		**0,047**		0,632	
**Longitudinalidade**	**7,5 ± 1,0**	6,8 ± 1,6	7,5 ± 0,9	7,3 ± 1,3	7,3 ± 1,3	7,4 ± 1,1	**7,7 ± 0,9**	7,1 ± 1,4
***p***	**0,008**		0,271		0,401		**< 0,001**	
**Integração do cuidado**	8,4 ± 1,6	8,1 ± 1,5	8,3 ± 1,8	8,3 ± 1,4	**7,8 ± 1,6**	8,5 ± 1,5	8,5 ± 1,6	8,1 ± 1,6
***p***	0,640		0,901		**0,021**		0,236	
**Sistemas de informação**	**8,4 ± 1,3**	7,5 ± 1,6	8,5 ± 1,2	8,2 ± 1,5	8,3 ± 1,6	8,3 ± 1,3	**8,7 ± 1,1**	7,8 ± 1,5
***p***	**< 0,001**		0,079		0,964		**< 0,001**	
**Serviços disponíveis**	6,0 ± 1,8	6,2 ± 1,7	5,6 ± 1,8	**6,4 ± 1,7**	6,4 ± 1,5	6 ± 1,8	6 ± 1,9	6,2 ± 1,6
***p***	0,684		**0,004**		0,082		0,351	
**Serviços prestados**	6,5 ± 1,7	6,2 ± 2,0	6,4 ± 1,9	6,6 ± 1,7	6,5 ± 1,4	6,5 ± 1,9	6,4 ± 1,7	6,6 ± 1,8
***p***	0,362		0,351		0,870		0,654	

*HAS - hipertensão arterial sistêmica.*

Pessoas idosas com DM demonstraram escores mais baixos no atributo de serviços disponíveis (5,6 ± 1,8) em comparação com pessoas sem DM (6,4 ± 1,7), com *p* = 0,004. Escores mais baixos também foram observados em pessoas idosas com artrite nos atributos de acesso ao primeiro contato (acessibilidade) (6,1 ± 0,8) e coordenação (integração de cuidados) (7,8 ± 1,6), com *p* = 0,047 e *p* = 0,021, respectivamente. Não obstante, pessoas com HAS avaliaram melhor os serviços de acesso ao primeiro contato (utilização) (9,1 ± 1,4), longitudinalidade (7,7 ± 0,9) e coordenação (sistemas de informação) (8,7 ± 1,1), com *p* = 0,041 para o primeiro atributo e *p* < 0,001 para os demais.

## DISCUSSÃO

Os resultados deste estudo revelam, em relação à caracterização sociodemográfica dos participantes da pesquisa, o predomínio de pessoas idosas do sexo feminino, com idade variando entre 60 e 90 anos. Houve maior frequência entre 60 e 69 anos, casadas, de baixa escolaridade e baixa renda familiar. Esses resultados estão em consonância com outras pesquisas nacionais que abordam a caracterização de pessoas idosas usuárias da APS^(15-17)^.

Os escores médios dos atributos, de modo geral, foram considerados satisfatórios. No entanto, o atributo essencial primeiro contato e seus componentes, como acessibilidade e utilização, evidenciou fragilidade, com escore médio pouco abaixo do valor de referência em relação à acessibilidade. A acessibilidade na saúde se refere a aspectos estruturais e à facilidade de acesso aos serviços, considerando fatores geográficos e organizacionais, como horários de funcionamento, agendamento de consultas e uso de ferramentas de comunicação^(4)^. Disponibiliza à população acesso aos serviços de saúde com eficiência no primeiro contato^(18)^, garantindo-lhe atendimento de forma oportuna. A avaliação deste atributo mostra dificuldades dos usuários em acessar e utilizar os serviços de saúde. Já a subdivisão utilização, que reportou alto escore, está vinculada ao uso do serviço pelos usuários a cada novo problema de saúde e para cada novo episódio de um problema prévio^(7)^. Resultados semelhantes foram encontrados em pesquisas realizadas com usuários adultos para avaliar o desempenho da APS, com o resultado de alto escore do componente utilização^(19,20)^.

Por meio de um alto escore médio, verificou-se a presença do atributo longitudinalidade nas ESFs. Estudo desenvolvido para avaliar a qualidade da APS na percepção de pessoas idosas corrobora esses dados, evidenciando avaliação satisfatória do atributo^(10)^. O atributo longitudinalidade infere a presença de uma fonte constante de atenção e de seu uso ao longo do tempo, sendo crucial para o fortalecimento de uma relação interpessoal entre usuários e equipe de saúde, refletindo a confiança mútua e o vínculo, promovendo assistência de qualidade que perpassa a resolução de apenas problemas imediatos^(4)^.

Estudo transversal envolvendo adultos usuários da APS em 32 municípios da 4ª Coordenadoria Regional de Saúde do RS (4ª CRS), utilizando o *PCATool*, revelou um escore médio insatisfatório no atributo longitudinalidade. Entretanto, observou-se que esse atributo foi mais bem avaliado em municípios com menor população, menor Índice de Desenvolvimento Humano e maior cobertura da ESF^(21)^. Santa Maria é a sede da 4ª CRS, com população estimada de 271.735 habitantes^(22)^. Na programação anual de saúde 2024 do município, há o objetivo de ampliar, através do cadastramento dos usuários pelas equipes de referência, a cobertura de 70% da população^(23)^. Contudo, até o momento, não foram identificadas informações públicas que confirmem a efetiva implementação ou alcance dessa meta.

O atributo essencial coordenação da atenção foi considerado alto em seus dois componentes - integração de cuidados e sistemas de informação -, coincidindo com pesquisa similar realizada com 345 pessoas idosas que obteve na análise dos resultados altos escores no que tange à integração de cuidados e sistema de informações. Ribeiro e Cavalcanti^(24)^ postulam que coordenar o cuidado envolve engajamento em atividades que garantam a prestação de cuidados individualizados e abrangentes, com foco na continuidade do processo de cuidado, e que obstáculos enfrentados pela APS na coordenação do cuidado incluem a necessidade de superar a fragmentação da rede, a escassez de vagas para especialistas, a falta de integração do prontuário eletrônico, a carência de qualificação profissional e a falta de reconhecimento do papel da APS pelos demais serviços de saúde.

No atributo integralidade, dividido em serviços disponíveis e serviços prestados, os escores encontrados foram, respectivamente, 6,9 e 6,1, sendo, assim, um escore considerado satisfatório e um pouco inferior ao valor de referência esperado. Estudo realizado em municípios do RS, que buscou avaliar o atributo da integralidade em serviços da APS, mostrou na análise do atributo resultados insatisfatórios. No entanto, o componente de serviço disponível obteve uma avaliação mais positiva em comparação com o componente serviço prestado^(25)^. Revisão sistemática sobre a integralidade na APS em diversos países apontou uma insatisfação generalizada com o nível de orientação à APS neste atributo, tanto nos serviços disponíveis quanto nos prestados. Ressalta-se a importância de avaliar a qualidade dos serviços e de ajustar e flexibilizar os serviços disponíveis para melhor atender às demandas e/ou encaminhar adequadamente os usuários^(26)^. Além disso, recomenda-se um apoio mais significativo das instituições de saúde e de seus gestores para melhorar as estruturas físicas, tecnológicas e de pessoal, incluindo capacitações contínuas para os profissionais de saúde^(26)^.

Ao realizar o escore essencial, que é a soma dos escores médios dos atributos essenciais, dividido pelo número de componentes, temos um valor satisfatório de 7,5, representando, de modo geral, a presença e extensão dos atributos essenciais nas ESFs do município. Pesquisa realizada em Manaus, Amazonas, com *PCATool* igualmente revelou o escore essencial alto/satisfatório^(27)^. No entanto, valores de referência abaixo do preconizado foram apresentados em pesquisas que utilizaram o *PCATool* para avaliação da APS, mostrando que nem todos os serviços de saúde estão norteados pelos atributos essenciais, necessitando de avaliações contínuas para melhoria da atenção à saúde nos serviços das ESFs^(18,20,28)^.

Ao analisar a associação entre variáveis sociodemográficas e os escores dos atributos essenciais, observa-se que as pessoas idosas do sexo masculino apresentam avaliações melhores em relação à acessibilidade, longitudinalidade e serviços prestados, quando comparados às médias das respostas das pessoas idosas do sexo feminino. Esses resultados diferem de outras pesquisas semelhantes, que indicam uma melhor avaliação dos atributos essenciais pelas mulheres^(29,30)^. Estudo sobre como os usuários do sexo masculino avaliam o acesso de primeiro contato na APS avaliou este aspecto como pouco orientado referente à acessibilidade, apresentando um escore médio baixo. Entretanto, o estudo não aborda a avaliação dos atributos relacionada à divisão por faixa etária^(31)^. Muitas pessoas idosas lidam com doenças crônicas, favorecendo um contato mais contínuo com os serviços de saúde^(32)^. Isso pode fortalecer a relação com tais serviços, aumentando sua familiaridade e contribuindo para uma avaliação positiva do atributo e das subdivisões mencionadas.

Observa-se, através dos resultados, que são necessárias melhorias, especialmente visando qualificar a atenção às mulheres idosas, que são as principais usuárias dos sistemas de saúde e têm necessidades de saúde específicas^(33)^. Além disso, a subdivisão acessibilidade apresentou fragilidades de modo geral, e especialmente no que se refere à acessibilidade das mulheres idosas, é necessário garantir um acesso de qualidade, com atendimento apropriado às suas especificidades.

Pessoas idosas na faixa etária de 60 a 69 anos avaliaram positivamente a coordenação dos sistemas de informação em comparação às pessoas idosas da faixa etária de 70 a ≥ 80 anos. O componente sistema de informações demonstra ser preciso quando o usuário vai ao serviço de saúde apresentar registros de saúde ou informações de um atendimento prévio^(19)^.

Pode-se afirmar que a longevidade no Brasil está em crescimento significativo, exigindo atenção especial para essa população. Nesse contexto, os sistemas de informação desempenham um papel crucial na estruturação e gestão dos dados de saúde dos usuários, para facilitar a implementação de estratégias tanto individuais quanto coletivas^(34)^ e disponibilizar informações do usuário na rede de serviços. Em outras palavras, ter um prontuário que contenha seus dados em todos os pontos da rede é fundamental para a coordenação do cuidado em saúde. O acesso às informações de saúde é um direito do usuário, sendo o registro no prontuário parte essencial desse direito^(35)^. Masochini *et al*.^(16)^ reportam que muitas pessoas idosas ainda desconhecem a possibilidade de acesso ao prontuário para o usuário consultá-lo, demonstrando a relevância de comunicar à pessoa idosa sobre esse direito.

Tratando-se de renda familiar, as pessoas idosas com renda igual ou superior a três salários mínimos também demonstraram uma melhor avaliação do atributo integralidade. Este resultado se assemelha a estudo anterior que utilizou o mesmo instrumento, indicando uma associação positiva entre renda mais alta e uma avaliação melhor da APS^(29)^. Outra pesquisa encontrou uma associação positiva entre o acesso ao primeiro contato e a coordenação da atenção entre pessoas idosas em situação de vulnerabilidade (baixa renda, áreas rurais e idade avançada), sugerindo, então, um progresso significativo na busca por equidade no acesso e qualidade dos serviços de saúde^(11)^. Portanto, é preciso concentrar esforços para efetivar a integralidade de modo equitativo à população idosa, garantindo, assim, a integralidade aos mais economicamente vulneráveis.

A maioria dos participantes do estudo apresentou comorbidades, sendo as mais prevalentes a HAS, seguida por DM e artrite/artrose, que são DCNTs prevalentes entre as pessoas idosas no Brasil^(24)^. Evidenciou-se, pela análise dos achados, que as pessoas idosas com comorbidades avaliaram melhor os atributos de longitudinalidade e o componente sistema de informações. A boa avaliação também foi observada em estudo com usuários adultos, indicando que aqueles com DCNTs avaliam os serviços de saúde de forma mais positiva^(29)^. Esta pesquisa pondera que a avaliação favorável decorre do maior uso desses serviços por esses usuários^(29)^. Já estudo realizado em Belo Horizonte, Minas Gerais, traz achados divergentes, mostrando a prevalência de DCNTs associada a uma percepção desfavorável do desempenho da APS nos atributos^(30)^. Destaca-se que a APS desempenha um papel crucial no cuidado das DCNTs e no controle dos fatores de risco, realizando ações para prevenir doenças, promover a saúde e minimizar danos.

A presente pesquisa encontrou uma associação significativa entre as pessoas idosas hipertensas e uma melhor percepção em aspectos da APS, como o componente utilização, o atributo longitudinalidade e o componente sistema de informações. Este achado corrobora estudo anterior realizado com adultos usuários, que também destacou uma avaliação superior da APS por parte de usuários com HAS e DM^(29)^. No entanto, outra pesquisa indica associação entre as pessoas idosas hipertensas em relação à coordenação do cuidado comparada às pessoas idosas sem essa condição^(30)^. Além disso, estudo realizado em Fortaleza, Ceará, revelou que ter HAS ou um diagnóstico de saúde mental pode estar associado a uma menor percepção da integralidade dos serviços disponíveis, já que pessoas com doenças crônicas, devido a uma maior utilização da APS, podem estar mais expostas a experiências negativas, o que pode resultar em uma perspectiva mais crítica em relação à atenção à saúde recebida^(20)^. Tendo em vista que o processo de envelhecimento da população tem impulsionado um significativo aumento das DCNTs^(36,37)^, torna-se indispensável o monitoramento das ocorrências das DCNTs para avaliar políticas públicas eficazes e melhorar o planejamento de saúde. Isso demanda uma abordagem renovada na APS, visando promover a saúde, prevenir mortes evitáveis e elevar a qualidade de vida dos pacientes afetados^(36)^. A qualidade dos cuidados na APS é essencial para enfrentar, de modo eficaz, as DCNTs, melhorando a saúde e qualidade de vida da população afetada.

No estudo, a maioria dos resultados da avaliação dos atributos essenciais da APS, pela percepção das pessoas idosas, foi satisfatória, com exceção da acessibilidade, a qual é um componente fundamental para o acesso ao primeiro contato. Destaca-se, portanto, a importância de melhorar aspectos como horários adequados para atendimento, comunicação eficiente, oferta de dias alternativos, organização no agendamento de consultas, entre outros. Isso evidencia um desafio para a gestão dos serviços de saúde e adequações do processo de trabalho das equipes de saúde^(6)^.

### Limitações do estudo

A aplicação do instrumento se restringiu às pessoas idosas que têm acesso à ESF, excluindo uma parte significativa da população de pessoas idosas que enfrentam barreiras de acesso^(38)^. A participação dessas pessoas poderia oferecer perspectivas distintas para avaliação dos atributos essenciais da APS, especialmente em relação à acessibilidade. Além disso, a falta de estudos específicos para uso do instrumento com pessoas idosas^(39)^ dificultou uma análise mais detalhada sobre os dados encontrados. Pode-se destacar também que a utilização de amostra não probabilística compromete a representatividade dos participantes, e pode limitar a generalização dos resultados para outras populações ou contextos semelhantes. Deve-se considerar também que o instrumento utilizado foi concebido para aplicação em adultos em geral sem abordar as particularidades relevantes para pessoas idosas.

### Contribuições para a área da enfermagem, saúde ou políticas públicas

Os achados demonstrados pela utilização do *PCATool* na avaliação da APS entre as pessoas idosas pode oferecer a oportunidade de aprimorar o cuidado a essa população, qualificando o atendimento de saúde à pessoa idosa na APS. Não obstante, contribui para aprimorar o conhecimento científico sobre o tema.

## CONCLUSÕES

Este estudo avaliou a qualidade da APS sob a perspectiva de 249 idosos cadastrados em ESF no município de Santa Maria. Os resultados indicaram escores globalmente satisfatórios para os atributos essenciais da APS, com exceção do acesso de primeiro contato e da integralidade, que apresentaram desempenho inferior. Identificaram-se associações estatisticamente significativas entre variáveis sociodemográficas (sexo, idade e renda) e presença de comorbidades com determinados atributos avaliados, sugerindo que essas características influenciam a percepção da qualidade do cuidado. Apesar da avaliação global positiva, persistem desafios relacionados à acessibilidade, integralidade e oferta de serviços, os quais demandam intervenções estratégicas. Esses achados destacam a necessidade de considerar as especificidades sociodemográficas e clínicas da população idosa no planejamento e na qualificação da APS, a fim de promover um cuidado mais equitativo e responsivo às suas necessidades.

## Data Availability

Os dados de pesquisa estão disponíveis no corpo do artigo.
